# Female Sex and Outcomes after Endovascular Aneurysm Repair for Abdominal Aortic Aneurysm: A Propensity Score Matched Cohort Analysis

**DOI:** 10.3390/jcm10010162

**Published:** 2021-01-05

**Authors:** Christian-Alexander Behrendt, Thea Kreutzburg, Jenny Kuchenbecker, Giuseppe Panuccio, Mark Dankhoff, Konstantinos Spanos, George Kouvelos, Sebastian Debus, Frederik Peters, Tilo Kölbel

**Affiliations:** 1 Research Group GermanVasc, Department of Vascular Medicine, University Heart and Vascular Center UKE Hamburg, University Medical Center Hamburg-Eppendorf, 20246 Hamburg, Germany; t.kreutzburg@uke.de (T.K.); j.kuchenbecker@uke.de (J.K.); s.debus@uke.de (S.D.); f.peters@uke.de (F.P.); 2 German Aortic Center Hamburg, Department of Vascular Medicine, University Heart and Vascular Center UKE Hamburg, University Medical Center Hamburg-Eppendorf, 20246 Hamburg, Germany; g.panuccio@uke.de (G.P.); spanos.kon@gmail.com (K.S.); tilokoelbel@googlemail.com (T.K.); 3 DAK-Gesundheit, 22788 Hamburg, Germany; mark.dankhoff@dak.de; 4 Department of Vascular Surgery, University Hospital of Larissa, Faculty of Medicine, School of Health Sciences, University of Thessaly, 41334 Larissa, Greece; geokouv@gmail.com

**Keywords:** outcome research, gender differences, healthcare research, abdominal aortic aneurysm, endovascular techniques

## Abstract

Objective: Previous studies have showed a potential disadvantage of female patients who underwent abdominal aortic aneurysm (AAA) repair. The current study aims to determine sex-specific perioperative and long-term outcomes using propensity score matched unselected nationwide health insurance claims data. Methods: Insurance claims from a large German fund were used, covering around 8% of the insured German population. Patients who underwent endovascular aortic repair (EVAR) for intact AAA from 1 January 2011 to 30 April 2017 were included in the cohort. A 1:2 female to male propensity score matching was applied to adjust for confounding variables. Perioperative and long-term outcomes after 5 years were determined using matching and regression methods. Results: Among a total of 3736 patients (19.3% females, mean 75 years) undergoing EVAR for intact AAA, we identified 1863 matched patients. Before matching, females were more likely to be previously diagnosed with hypothyroidism, electrolyte disorders, rheumatoid disorders, and depression, while males were more often diabetics. In the matched sample, 23.4% of the females and 25.8% of the males died during a median follow-up of 776 and 792 days, respectively. Perioperatively, females were more likely to exhibit acute limb ischemia (5.3% vs. 3.2%, *p* = 0.031) and major bleeding (22.0% vs. 15.9%, *p* = 0.001) before they were discharged to rehabilitation (5.5% vs. 1.5%, *p* < 0.001) when compared to males. No statistically significant difference in perioperative (odds ratio 1.12, 95% CI 0.54–2.16) or long-term mortality (hazard ratio 0.91, 95% CI 0.76–1.08) was observed between sexes. This was also true regarding aortic reintervention rates after 1 year (2.0% vs. 2.9%) and 5 years (10.9% vs. 8.1%). Conclusion: The current retrospective matched analysis of insurance claims revealed high early access-related morbidity in females when compared to their male counterparts. Short-term or long-term survival and reintervention outcomes were similar between sexes.

## 1. Introduction

The scientific discussion around sex disparities and female patient disadvantage in cardiovascular medicine has been ever-present for decades [1]. There is growing evidence for different symptoms in females vs. males suffering from peripheral arterial occlusive disease (PAOD) [2,3,4], coronary artery disease [5], atrial fibrillation [6,7], and heart failure [8]. As for PAOD, previous publications suggested a female sex disadvantage with regard to invasive and pharmacological treatment, emphasizing the urgent need for sex-specific research [3,4,9,10]. However, it is known that randomized controlled trials include fewer females when compared with real-world cohorts. Moreover, most valid societal practice guidelines on diseases of the peripheral arteries to date contain very few sex-specific recommendations [11,12,13,14].

The open surgical approach (OSR) and endovascular (EVAR) treatment of abdominal aortic aneurysm (AAA) has been illuminated by numerous observational studies during recent decades [15,16,17,18]. While a different prevalence and risk profile between sexes are common knowledge, research on technical and anatomical details and outcomes remains scarce [19,20]. For complex endovascular aortic repair using fenestrated and branched EVAR, a higher rate of bleeding requiring transfusion and spinal cord ischemia, was previously observed in female patients. The occurrence of these devastating events was associated with worse survival in the short- and long-term follow-up [21,22].

The aim of the current study is to determine sex-specific perioperative and long-term outcomes of EVAR for intact AAA using propensity score matched unselected nationwide health insurance claims data. The research hypothesis is that direct and indirect disparities exist between sexes.

## 2. Methods

### 2.1. Study Design

The health insurance claims data of Germany’s third largest insurance provider, DAK-Gesundheit (DAK-G) include the outpatient and inpatient medical care provided to a subset of approximately 6.5 million German citizens (8% of the insured German cohort). In Germany (data for 2017), approximately 72 million inhabitants are insured by statutory health insurance; another 10.5 million inhabitants are insured by other types of insurance (e.g., private health insurance). At each patient contact (e.g., inpatient treatment or regular surveillance) in outpatient facilities and legally endorsed hospitals, a dataset was created and submitted to the health insurance fund. DAK-G data have frequently been used for studies before [21,22,23]. The DAK-G cohort includes nationally generalizable data with comparable sex and age distribution and has been validated before. In addition, the health insurance funds in Germany charge the Medical Service of the Health Funds to perform a random and risk-based validation (quality assurance) of data.

### 2.2. Study Variables

The diagnoses and comorbidities routinely collected in health insurance claims data follow the commonly accepted international standard for reporting diseases and health conditions using the World Health Organization (WHO) International Classification of Diseases in its 10th revision of the German Modification (ICD-10-GM) and Operations and Procedures Codes (OPS) as a German adaptation of the International Classification of Procedures in Medicine (ICPM) by the WHO.

### 2.3. Inclusion Criteria

All statutory health-insured patients with at least one hospital stay between 1 January 2011 and 30 April 2017 for intact AAA and Operation and Procedure Classification System (OPS) codes for EVAR of the abdominal infrarenal aorta (endovascular implantation of stentgraft without fenestration or branches: 5-38a*, 8-842*, 8-84a/b*) were investigated.

### 2.4. Statistical Analysis

Statistical analyses and data reporting are in accordance with the statistical and data reporting guidelines on good practice in secondary data analysis and RECORD [24]. To adjust for confounding variables in this non-randomly assigned cohort, we performed a 2:1 propensity score matching (standardized mean differences > 0.1) for males and females using the Elixhauser comorbidity groups, the van Walraven score, and length of hospital stay (days). Multivariable regression models were used to determine the independent association of sex with in-hospital and long-term survival using a logistic and Cox regression approach. The unmatched models included age, female sex, van Walraven score, and length of hospital stay for adjustment. Kaplan–Meier survival curves were used to determine long-term survival, and a log rank test was used. Patients with unknown survival time due to the end of the study period were right censored after five years. No adjustment for multiple hypothesis testing was applied. Statistical significance was defined as a *p* value < 0.05.

All statistical analyses were performed with R version 4.0.3 (The R Foundation for Statistical Computing, Vienna, Austria). Visualization was performed with Adobe Illustrator version 24.1.2 (Adobe, San Jose, CA, USA).

## 3. Results

We identified 3736 patients (17.3% females) who underwent EVAR for intact AAA between January 2011 and April 2017. The mean age was comparable between females and males (74.88 ± 8.30 vs. 74.45 ± 8.03 years). Females were more often diagnosed with hypothyroidism (22.6% vs. 9.4%), fluid and electrolyte disorders (41.1% vs. 28.0%), rheumatoid disorders (5.1% vs. 2.6%), and depression (8.2% vs. 4.4%). The prevalence of diabetes was higher in male patients when compared to their female counterparts (21.4% vs. 16.9%). Female patients had a longer length of hospital stay when compared to males (9 vs. 8 days) and were discharged to rehabilitation more often (5.4% vs. 1.2%). The perioperative mortality after 30 days was 2.5% in female patients vs. 2.2% in male patients. After 90 days, the mortality was 5.1% vs. 3.6% (unmatched results not shown in tables, all differences: standardized mean differences > 0.1).

### 3.1. Propensity Score Matched Cohort

In total, 1863 patients (34.1% females) were included in a propensity score matched sample. One hundred and forty-nine (23.4%) of the females and 316 (25.8%) of the male patients died during the entire follow-up (median 776 vs. 792 days). The baseline characteristics of the propensity score matched cohort are presented in Table 1.

### 3.2. Perioperative Outcomes in the Propensity Score Matched Cohort

The mortality rates of female vs. male patients were similar during the in-hospital stay (1.7% vs. 1.6%, *p* = 1.000), after 30 days (2.4% vs. 3.0%, *p* = 0.504), and after 90 days (5.0% vs. 4.8%, *p* = 0.922). Females were more often discharged to rehabilitation (5.5% vs. 1.5%, *p* < 0.001), and they exhibited acute limb ischemia (5.3% vs. 3.2%, *p* = 0.031) and major bleeding (22.0% vs. 15.9%, *p* = 0.001) more often when compared to males (Table 2). Within 30 days, 4.0% (95% CI 2.4% to 5.6%) of females and 3.6% (95% CI 2.6% to 4.6%) of males were readmitted to a hospital.

In adjusted analysis, older age (Odds ratio, OR 1.046, 95% CI 1.006–1.089), higher van Walraven score (OR 1.077, 95% CI 1.049–1.104), and longer length of hospital stay (OR 1.019, 95% CI 1.003–1.033) were associated with higher odds for in-hospital mortality. Female sex did not add significantly to the model (Table 3).

### 3.3. Long-Term Survival in the Propensity Score Matched Cohort

**Figure 1:**
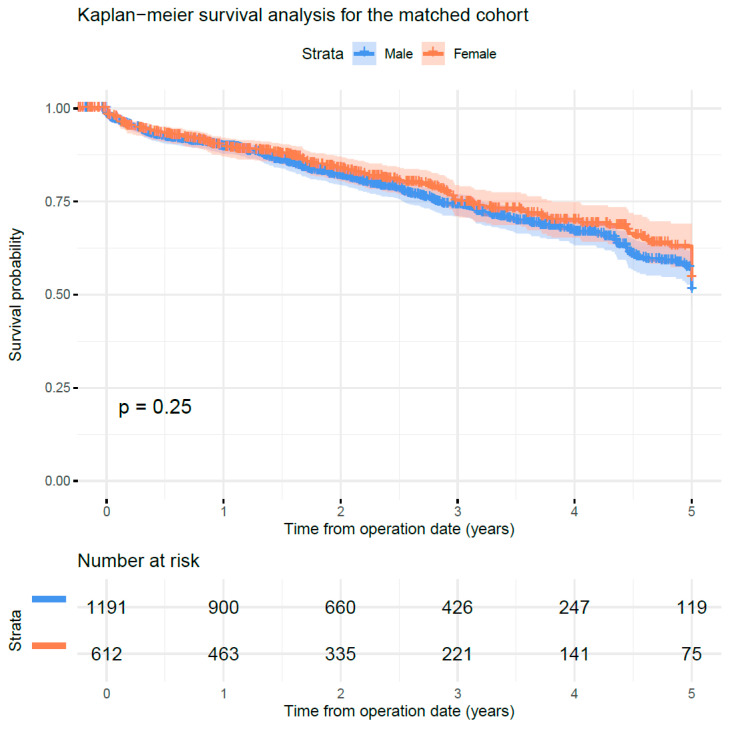
Kaplan–Meier survival analysis for this propensity score matched retrospective analysis of 1863 matched patients who underwent endovascular repair (EVAR) for intact abdominal aortic aneurysm between 2011 and 2017 by female vs. male sex. *p*-value for log rank test.

The Kaplan–Meier survival curves and the result of the log rank test (*p* = 0.250) are presented in Figure 1. No statistically significant mortality differences between both sexes were observed during the five-year follow-up.

Within 1 year after the procedure, 2.0% (95% CI 0.8% to 3.2%) of females and 2.9% (95% CI 1.9% to 3.6%) of males exhibited any reoperation, while these rates increased to 10.8% (95% CI 6.2% to 15.1%) and 8.1% (95% CI 5.8% to 10.3%) after 5 years, respectively.

In adjusted analysis, older age (Hazard ratio, HR 1.055, 95% CI 1.045–1.064), higher van Walraven score (HR 1.054, 95% CI 1.047–1.061), and longer length of hospital stay (HR 1.017, 95% CI 1.012–1.021) were associated with higher risk for long-term mortality. Female sex did not add significantly to the model (Table 3).

## 4. Discussion

In this large-scale retrospective analysis of propensity score matched health insurance claims, we observed only marginal differences with regard to the sex-specific risk profile of patients who underwent EVAR for intact AAA. After matching for the most relevant confounders, females exhibited perioperative acute limb ischemia and major bleeding more often. Furthermore, they were discharged to rehabilitation more frequently when compared to their male counterparts. Interestingly, no significant association between female sex and survival or reintervention rate was observed in the short- and long-term follow-up after proper matching was applied. This central finding appears to conflict with previous evidence from observational research.

In a recently published systematic review, Stoberock et al. identified some 15 observational studies addressing sex disparities in AAA repair, covering a period from 1976 to 2016. The authors found a higher complication rate and longer hospital stay after OSR and EVAR for AAA in females, as well as worse long-term survival. They concluded that guidelines should take sex-specific treatment strategies into account [25]. Liu et al. most recently included 36 cohorts in a meta-analysis. The pooled results showed that female sex was associated with a significantly increased risk of in-hospital, 30-day, and long-term all-cause mortality. Besides these interesting findings that were not confirmed by the current study, the authors also found an increased prevalence of acute limb ischemia in females (OR 2.44, 95% CI, 1.73–2.43, *p* < 0.001), confirming our findings [26].

Deery et al. used the targeted vascular module of the National Surgical Quality Improvement Program (NSQIP) to identify patients undergoing EVAR or OSR for intact AAA from 2011 to 2014. They identified 6611 patients (19% females) and found higher 30-day mortality (3.2% vs. 1.2%) and major complication rates (9.6% vs. 5.8%) after elective EVAR in females. The higher rates of transfusions (16.0% vs. 8.4%) and leg ischemia (2.8% vs. 1.1%) were confirmed by the current study, although the overall rates were considerably higher in claims data when compared to the clinical registry [27]. The occurrence of acute limb ischemia and perioperative bleeding events may be more related to morphological differences between sexes, like smaller iliac access vessels, when compared to mortality alone. Further emphasizing this hypothesis, striking sex differences were observed in complex endovascular aortic interventions, where differences in access vessel diameter and blood volume may play a more important role [21,22,28]. Supporting this hypothesis, Etkin et al. reported their experience at a large tertiary medical center between 2003 and 2013. They concluded that EVAR can be successfully performed in patients with bilateral small iliac arteries, and that adjunctive procedures might increase the technical success rate [29]. Khashram et al. conducted a systematic review and meta-analysis of 51 publications to identify risk factors validly predicting long-term survival following elective AAA repair. Female sex and the association with late survival were assessed by 16 very low-quality studies including 49,653 patients with serious inconsistency. Females exhibited a higher overall mortality than males (HR 1.15, 95% CI 1.07–1.27) [30].

Using data from Medicare beneficiaries and a national clinical registry in the United States, the Vascular Quality Initiative, Ramkumar et al. analyzed 16,386 patients who underwent OSR and EVAR from 2003 to 2015. After EVAR, females were 13% more likely to die than males [31]. Bulder et al. analyzed 12,907 (17% female) patients who underwent elective AAA repair between 2001 and 2015 in Sweden. The authors observed a persistently more compromised 4-year survival of female patients when compared to males. Interestingly, death with a cardiovascular cause was more common in females, which again emphasizes the importance of surveillance and best medical treatment [19].

Since most previous studies revealed an association between female sex and worse survival, the question arises of how the current findings can be interpreted. Aside from methodological differences between observational studies (e.g., adjustment for residual confounding variables, definition of core variables), the peculiarities between included target populations must be considered. For instance, females in Germany have a 4.7-year longer life expectancy than males, while this difference was only 3.1 years in Sweden (in 2017). The fact that an observed trend towards higher 90-day mortality together with a higher risk for perioperative complications and discharge to rehabilitation in female patients of the current study did not materialize a lower long-term survival of female patients may be caused by the generally longer life expectancy of females in the German population. Having that said, a similar survival between sexes may imply worse outcomes in females [30]. Interestingly, there is evidence for wide variations between countries, databases, and numerous external factors associated with the reimbursement system [10,32]. To date, these methodological aspects appear underrepresented in the international discussion.

Notably, in a retrospective observational study of 934 consecutive patients (13% females) who underwent EVAR between 1997 and 2011 at a tertiary center in the United States, the authors found that female sex was associated with complications and reinterventions at a significant level, but not mortality [33]. The current study confirmed these interesting findings while adding a multicentric perspective.

Interestingly, female patients were more often discharged to rehabilitation when compared to males, which confirmed findings from a previous study on complex endovascular aortic repair [34]. In Germany, patients can request a rehabilitation after the index hospital stay. Following this request, the treating physicians prepare an application including the medical reasons and potential benefit with regard to the specific circumstances. There are complex criteria that are evaluated for each specific application. With regard to open surgical or endovascular aortic repair, rehabilitation is usually recommended and would most likely be granted.

### This Study Has Limitations

The retrospective observational design of the current study does only allow us to derive associations and to generate hypotheses that should be addressed by prospective studies in the future. Since patients’ sex cannot be randomized, clinical and administrative registries will play an important role. However, the international vascular community would be well advised to further develop methods to improve the external and internal validity of data. Since the diameter and tortuosity of access vessels as well at the specific type of endoprosthesis was not available in the current study, these possible confounders remain unaddressed. Certainly, there is evidence for an impact of those variables, especially concerning short-term results [20,35]. Moreover, the aspect regarding patients who have been turned down for invasive AAA repair remains another largely unsolved issue in clinical and administrative registries that follow a procedure-related approach. It can be suspected that the turn-down rate for elective EVAR is different between sexes, which introduces an important bias. Lastly, no valid information was available on smoking and optimal pharmacological treatment to adjust for these confounders.

The development of societal guidelines was mainly based on male populations included in historical randomized trials. Even though the relationship between female sex and worse survival remains conflicting in the existing literature, there is growing evidence for higher complication rates after certain procedures in females that should be further illuminated by future studies. The increased number of sex-specific recommendations of international guidelines, as in Wanhainen et al., is commendable and an important tool to establish sex-specific treatments [36].

Future randomized controlled trials should at least try to represent a female to male ratio that is comparable to real-world cohorts.

## 5. Conclusions

The current retrospective matched analysis of insurance claims revealed higher rates of acute limb ischemia and major bleeding after EVAR for AAA in females when compared to their male counterparts. No statistically significant differences were observed with regard to short-term or long-term survival between both sexes.

## Figures and Tables

**Table 1:** Baseline characteristics of the propensity score matched cohort in this retrospective observational study of health insurance claims.

	Female Sex	Male Sex	*p*-Value	SDIFF
Number, *n*	636	1227		
Median follow-up, days (IQR)	776 (326–1375)	792 (346–1348)	0.925	0.008
Mean age, years (SD)	74.88 (8.28)	75.06 (7.92)	0.646	0.022
Congestive heart failure, *n* (%)	144 (22.6)	276 (22.5)	0.989	0.004
Cardiac arrhythmias, *n* (%)	164 (25.8)	325 (26.5)	0.787	0.016
Chronic pulmonary disease, *n* (%)	156 (24.5)	285 (23.2)	0.569	0.031
Diabetes, *n* (%)	109 (17.1)	210 (17.1)	1.000	0.001
Hypothyroidism, *n* (%)	138 (21.7)	227 (18.5)	0.112	0.080
Renal failure, *n* (%)	180 (28.3)	332 (27.1)	0.606	0.028
Liver disease, *n* (%)	25 (3.9)	45 (3.7)	0.877	0.014
Obesity, *n* (%)	72 (11.3)	142 (11.6)	0.932	0.008
Fluid and electrolyte disorders, *n* (%)	258 (40.6)	478 (39.0)	0.533	0.033
Van Walraven score 1–4, *n* (%)	158 (24.8)	326 (26.6)	0.699	0.058
Van Walraven score ≥5, *n* (%)	456 (71.7)	852 (69.4)		
Length of hospital stay, median (IQR)	9 (7–14)	8 (6–14)	0.032	0.004

SDIFF: Standardized mean difference. SD: Standard deviation. IQR: Interquartile range.

**Table 2:** Outcomes of the propensity score matched cohort in this retrospective observational study of health insurance claims.

	Female Sex	Male Sex	*p*-Value	SDIFF
Number, *n*	636	1227		
Overall mortality, *n* (%)	149 (23.4)	316 (25.8)	0.297	0.054
In-hospital mortality, *n* (%)	11 (1.7)	20 (1.6)	1.000	0.008
30-day mortality, *n* (%)	15 (2.4)	37 (3.0)	0.504	0.041
90-day mortality, *n* (%)	32 (5.0)	59 (4.8)	0.922	0.010
Transfer to another hospital, *n* (%)	12 (1.9)	37 (3.0)	0.197	0.073
Discharged to rehabilitation, *n* (%)	35 (5.5)	19 (1.5)	<0.001 ^#^	0.216
Acute respiratory insufficiency, *n* (%)	33 (5.2)	88 (7.2)	0.122	0.082
Pneumonia, *n* (%)	7 (1.1)	41 (3.3)	0.006 ^#^	0.153
Acute renal insufficiency, *n* (%)	16 (2.5)	36 (2.9)	0.710	0.026
Acute myocardial infarction, *n* (%)	7 (1.1)	8 (0.7)	0.451	0.048
Stroke or TIA, *n* (%)	12 (1.9)	26 (2.1)	0.870	0.017
Acute bowel ischaemia, *n* (%)	3 (0.5)	15 (1.2)	0.186	0.082
Acute limb ischemia, *n* (%)	34 (5.3)	39 (3.2)	0.031 ^#^	0.107
Major bleeding, *n* (%)	140 (22.0)	195 (15.9)	0.001 ^#^	0.157

SDIFF: Standardized mean difference. SD: Standard deviation. TIA: Transient ischemic attack. ^#^ denotes significant differences.

**Table 3:** Results of the multivariable logistic and Cox regression models.

	In-Hospital Mortality		Long-Term Mortality	
	Odds Ratio	95% CI	Hazard Ratio	95% CI
Older age	1.046 ^#^	1.006–1.089	1.055 ^#^	1.045–1.064
Higher van Walraven score	1.077 ^#^	1.049–1.104	1.054 ^#^	1.047–1.061
Length of hospital stay	1.019 ^#^	1.003–1.033	1.017 ^#^	1.012–1.021
Female sex (vs. male)	1.124	0.538–2.159	0.907	0.758–1.084

CI: Confidence interval. ^#^ denotes statistical significance.

## Data Availability

The data presented in this study are available on request from the corresponding author. The data are not publicly available due to legal restrictions.
